# Editorial: One health: the psychology of human-nature relationships for planetary and human wellbeing, volume II

**DOI:** 10.3389/fpsyg.2024.1386313

**Published:** 2024-03-26

**Authors:** Eric Brymer, Elizabeth Louise Freeman, Miles Richardson

**Affiliations:** ^1^Faculty of Health, Southern Cross University, Gold Coast Campus, QLD, Australia; ^2^Manna Institute, University of New England, Armidale, NSW, Australia; ^3^Sheffield Hallam University, Sheffield, United Kingdom; ^4^University of Derby, Derby, United Kingdom

**Keywords:** nature, health, one health (OH), human-nature relationships, ecological

In 2019 when we completed the first volume of this Research Topic we concluded that health service provision should consider how best to integrate nature and pointed to new upcoming notions, such as social prescribing, as possible means to capture these benefits at a community level. We also warned that the ‘nature as commodity or resource' way of thinking might lead to unforeseen and unwanted outcomes. We highlighted the human-nature relationship and that human health was dependent on healthy, biodiverse environments. As such, we called for interdisciplinary, non-reductionist, collaboration to a better understand how the human-nature relationship enhances mental health and wellbeing and to better design interventions and environments to support a high quality human-nature relationship (Brymer et al., 2019). We summed up the Research Topic with 6 recommendations: (1) green space close to home and work is important, (2) experiences with nature should be wide-ranging and across all seasons, (3) green spaces need to consider all biodiversity, (4) design our green spaces to encourage engagement and physical activity, (5) consider specialized therapeutic environments and contexts such as green prescriptions and (6) nature imagery and VR opportunities may be useful for some people.

A lot has happened since the publication of this Research Topic, not just in terms of research and practice but also in ways that have impacted our planet globally. Not least the onset of COVID-19 and various international conflicts. However, research examining the human-nature relationship and its connection to health has continued to grow as more and more studies show how different human conditions can be positively impacted by nature. At the same time, we are revealing the important impacts of different environment qualities that suggest we need to be mindful about what determines a high-quality health enhancing environment (e.g., Feng and Astell-Burt, 2022; Clark et al., 2023; Foley et al., 2023). While many studies have contributed to an increased pool of contexts where nature connection and contact seem to benefit human health in different ways, the field has not been without its critiques. We are learning that while nature has positive impacts on people a one-size-fits-all approach is limited. Scholars have called for a consistent understanding of what exposure to nature means and how best to measure it, a better understanding of the impacts of how long and how often we are exposed to nature as well as a call to better understand the nuances of the qualities of nature and the impact of these qualities on different people. Critics have also called for more appropriate frameworks that better explain the underlying mechanisms for how the human-nature relationship benefits health so that we can better provide appropriate environments and effectively design activities (Brymer et al., 2020; Jimenez et al., 2021; Lengieza et al., 2023). A call partially echoed in this Research Topic.

Nevertheless, it was comforting to find out that a Research Topic on the human-nature relationship still attracts 20 publications. We received papers examining the impact of social media (Xu et al.) to those examining surf and psychotherapy (Xu et al.; Meuwese et al.; Hinds). Submissions that dug deep into profound experiences (Mathers and Brymer) and those that examined nature-based physical activity (Jenkins et al.; Chambers and Poidomani). Papers that explored climate change (Whelan et al.; Kałwak and Weihgold; Galway and Beery) and other global issues (Masterson-Algar et al.) whilst other authors examined the implications of interventions (Passmore et al.; Ward et al.) in various contexts and environments. We had submissions that explored the application of particular traditional theoretical frameworks (Johnson et al.) as well as those that questioned traditional frameworks and proposed more evolutionary (Stoltz) and ecologically grounded perspectives (Brito et al.). Additionally, green social prescribing (Lawson et al.; Thomas et al.) was examined as a means to structure the health benefits of nature within a range of various contexts, discussing the value of virtual nature (Hsieh and Li) for certain needs and the implications of nature in urban environments and more constrained contexts such as Zoos (Rose and Riley).

As with the first volume, the broad range of submissions provided insights into the human-nature relationship and its implications for the health of people and the planet. For the most part, papers responded to the recommendations we made in the first volume and in many ways moved the field a little further along. However, this Research Topic also provides support for previous research that has questioned whether we are relying too much on critiqued perspectives to frame our studies and design our interventions (Brymer et al., 2020). Several themes emerged, reflecting the need for a holistic approach to research and collaboration that investigates the relationship between characteristics of the environment, individual and activities to address global challenges. Three prominent themes are (1) the importance of interdisciplinary efforts, (2) the role of global agendas as a bridge between diverse disciplines, and (3) the creation of networks to facilitate communication and cooperation.

Firstly, the publications in this Research Topic emphasize the significance of interdisciplinary efforts in addressing global challenges. It acknowledges the fragmentation of disciplines and ideologies in both sciences and other sectors, highlighting the necessity for strengthening collaborative approaches and more nuanced conceptual understandings. Collaborations between researchers, stakeholders, and policymakers from a wide range of disciplines is more likely to achieve working models that address local and global agendas effectively. By incorporating perspectives from disciplines such as (but not limited to) psychology, anthropology, health, sport, ocean and biological sciences, a multidisciplinary approach is advocated to navigate the intertwined nature of societal challenges.

Secondly, papers in this Research Topic recognize the important role of global agendas and local actions as a bridge between diverse disciplines. It asserts that global agendas can link various fields of study and serve as an overarching framework to engage in research that addresses complex societal challenges. This approach recognizes the interconnectedness of issues such as health, environment, and socio-economic dynamics. By considering global agendas as a unifying force, it becomes possible to accommodate the socio-economic and cultural differences among countries, fostering collaboration on an international scale and local level.

Thirdly, the Research Topic underscores the importance of creating networks to facilitate communication and cooperation. It highlights successful examples of bringing together researchers, stakeholders, and policymakers from different backgrounds under the umbrella of global agendas. By fostering communication between networks, the Research Topic provides some encouragement to the establishment of broad collaborations and the promotion of effective interdisciplinary research. These networks serve as platforms to exchange ideas, align efforts, and develop coordinated actions to tackle global health and environmental agendas for the next decades.

Overall, a number of recommendations for future research stem from this Research Topic.

## Recommendation 1

Develop openness to alternative frameworks to understand human nature relationships especially in relation to health and wellbeing benefits, a process that requires a paradigm shift to become mainstream. As researchers we have often relied on limited traditional theoretical frameworks (Brymer et al., 2020) to guide the design of research projects and interpret the findings from our studies. Papers from this Research Topic suggest that these traditional conceptualisations may not be able to fully explain how the human-nature relationship enhances health and wellbeing. It is perhaps imperative that we move away from using the same frameworks of thinking and doing the same thing over and over again and rather find new and more effective ways to encourage and understand the human-nature relationship. Practical implications of continuing a binary or narrow route of investigation could also mean that intervention design is relying on weak or even flawed frameworks for guidance. More effective frameworks are essential if we are to effectively design health enhancing environments in urban contexts or even to support effective intervention design and implementation in remote, rural or regional settings.

## Recommendation 2

It is important that we encourage multidisciplinary efforts and collaboration to address global challenges. For the most part experiences with nature enhance human health and wellbeing and the reciprocal process is that humans act in ways that enhance the health of nature. This seems to be a natural process for building health enhancing environments. However, climate change and extreme weather events seem to have provided challenges to this process. These are local and global challenges with the potential for considerable human and environment impact. It is perhaps imperative that we support collaborative multidisciplinary ways to explore and respond to these changes. Disciplines that do not often work closely together such as psychology and forestry or health and environment need to find ways to collaborate. It is perhaps no longer useful to focus on a siloed disciplinary approach to research or application.

## Recommendation 3

There is a need to explore working models and frameworks across countries and disciplines to address global agendas. Frameworks that work for one country or one discipline need to be carefully examined in this new global context. This is not to suggest that local needs are not important or that local needs might require an adaption of more global appreciations, more that we need to have a global frame for the future. Westernized approaches may seem to be very popular based on research findings but it may well be that more effective frameworks and interventions can be designed from indigenous ways of knowing, for example. Programs and interventions designed to facilitate health change and foster environmental stewardship in people should be encouraged and funded. Based on evidence and effective conceptual frameworks it seems clear that there needs to be an interdisciplinary global effort to facilitate stronger human-nature relationships and subsequently greater environmental stewardship and positive individual and collective climate action. Despite the incredible amount of research examining the reciprocal health and wellbeing benefits of the human-nature relationship we still seem to struggle to integrate these findings into policy, system and practice and link it to planetary wellbeing priorities in some instances, it would seem we actively work against any notion of system wide integration and societal change. However, this Research Topic and research in the broader space suggests that the benefits from such an integration would be wide reaching. We should encourage and find ways of supporting programs (e.g., green social prescribing, nature therapy, forest therapy) that are designed to facilitate direct experiences with nature for health and wellbeing and that can also lead to environmental stewardship and climate action where possible and appropriate. It is perhaps about time that nature-based solutions are embedded within health curriculum and planetary welfare agendas.

## Recommendation 4

While the international call to provide opportunities for children and young people to be experientially embedded in nature has been heard, research suggests that the benefits of nature are not limited to young people. It seems nature has the capacity to restore psychological function across the lifespan. Ensuring that we have enough quality nature spaces across all domains of human life (workplace, urban localities, schools and so on) is still vital. Perhaps even more so, in this second edition, there is a suggestion that providing appropriate opportunities for being physical, in relation to nature and greenspaces is important. Could it be that sport in the traditional appreciation of the word has been surpassed by nature-based physical activity in terms of health benefits? We need to explore beyond our need for safety to embrace affordances in less manicured natural environments that are more effective at facilitating thriving and flourishing. Humans have evolved in nature and are more likely to thrive when acting in relation to nature. At the same time, efforts to increase nature connection need to ensure that nature is not considered and used as a “commodity.” That programs and research move toward a more eco-centric direction rather than an anthropocentric one. This will help perpetuate the one health agenda and prioritize nature regeneration and therefore human health as well.

In summary, the papers in this Research Topic emphasize the need for a holistic and collaborative approach to address global challenges. The Research Topic advocates for multidisciplinary efforts, leveraging global agendas as a unifying framework, and establishing networks to foster communication and cooperation. By embracing these themes, researchers, policymakers, and stakeholders can work together to develop innovative solutions that transcend disciplinary boundaries and effectively address the interconnected nature of global challenges. However, integrated vision depends upon the human-nature relationship being seen as a mainstream topic, especially in Western societies where the fracture between humans and nature is still deep. This likely requires a paradigm shift to more ecological framework for understanding the human-nature relationship.

## Author contributions

EB: Writing—review & editing, Writing—original draft, Investigation, Conceptualization. EF: Writing—review & editing, Writing—original draft, Conceptualization. MR: Writing—review & editing, Writing—original draft, Conceptualization.
